# Tavis–Cummings Model with Moving Atoms

**DOI:** 10.3390/e23040452

**Published:** 2021-04-12

**Authors:** Sayed Abdel-Khalek, Kamal Berrada, Eied M. Khalil, Hichem Eleuch, Abdel-Shafy F. Obada, Esraa Reda

**Affiliations:** 1 Department of Mathematics and Statistics, College of Science, Taif University, P.O. Box 11099, Taif 21944, Saudi Arabia; sabotalb@tu.edu.sa (S.A.-K.); eiedkhalil@yahoo.com (E.M.K.); 2 Department of Physics, College of Science, Imam Mohammad Ibn Saud Islamic University (IMSIU), Riyadh 11432, Saudi Arabia; 3 The Abdus Salam International Centre for Theoretical Physics, Strada Costiera 11, 34151 Miramare-Trieste, Italy; 4 Department of Applied Physics and Astronomy, University of Sharjah, Sharjah 27272, United Arab Emirates; hichemeleuch@yahoo.fr; 5 Department of Applied Sciences and Mathematics, College of Arts and Sciences, Abu Dhabi University, Abu Dhabi 59911, United Arab Emirates; 6 Institute for Quantum Science and Engineering, Texas A&M University, College Station, TX 77843, USA; 7 Department of Mathematics, Faculty of Science, Al-Azhar University, Cairo 13759, Egypt; asobada@yahoo.com; 8 Department of Mathematics, Faculty of Education, Ain Shams University, Cairo 11566, Egypt; 12@imamm.org

**Keywords:** power-law potentials, entanglement, two qubits, statistical properties, concurrence, cat states, 03.67.-a, 03.65.Yz, 03.65.Ud

## Abstract

In this work, we examine a nonlinear version of the Tavis–Cummings model for two two-level atoms interacting with a single-mode field within a cavity in the context of power-law potentials. We consider the effect of the particle position that depends on the velocity and acceleration, and the coupling parameter is supposed to be time-dependent. We examine the effect of velocity and acceleration on the dynamical behavior of some quantumness measures, namely as von Neumann entropy, concurrence and Mandel parameter. We have found that the entanglement of subsystem states and the photon statistics are largely dependent on the choice of the qubit motion and power-law exponent. The obtained results present potential applications for quantum information and optics with optimal conditions.

## 1. Introduction

When the Jaynes–Cummings (JC) model was firstly proposed in 1963 [1,2], its practical significance was not clear, as it prescribes the ideal situation of the resonant interaction of a two-level atom with an electromagnetic field. A complete solvable quantum model of a qubit in a single-mode field was studied to examine the classical properties of spontaneous emission and to detect the presence of Rabi oscillations in the atomic excitation potentials of fields of sharply defined energy. In the 1980s, due to technical progress, the importance of this model increased significantly, since many of its predictions were confirmed experimentally [3,4,5]. It is worth noting that even though the JC model is simple and easy to implement, it still exhibits many physical effects, such as squeezing [6], Rabi oscillations [7,8], revivals and collapses [9,10,11,12], qubit–field entanglement [13,14], antibunching [15,16], and nonclassical states such as Fock states [17,18] and Schrödinger cat states [19]. The JC model was originally designed to describe the interaction of a single atom with a single-mode field, so it can be applied to various physical scenarios such as flux qubits [20], Josephson junctions [21,22] and Cooper-pair boxes [23]. This model can also be used in solid-state systems to characterize the coupling of qubits to resonator modes, considering, superconducting circuits [24,25,26,27,28,29] and quantum dot systems [30,31]. One of the most important generalizations of the JC model is the interaction of many atoms with the single-mode field presented by the Tavis–Cummings model, where *N* identical two-level atoms interacting with a single-mode of electromagnetic field at resonance has been studied [32]. Another important implementation of the JC model is the characterization of Rydberg blockaded atomic ensembles [33]. The detection and measure of nonclassical effects, phenomena that Maxwell’s equations cannot explain, are the main tasks of experimental and theoretical quantum optics. Over the last several decades, many of these effects have been intensively studied, such as entanglement [34], squeezing [35,36] and photon antibunching [37]. However, there exist other effects outside of this set, such as quantum correlations caused by the violation of the field intensity inequality [38,39].

Since the work published by Einstein et al. [40], entanglement has been considered one of the most prominent features of quantum mechanics and has attracted a great amount of interest. The quantum entanglement can be utilized for more than just testing and detecting the quantum nonlocality [41]; it also plays an important role in the science of quantum information, such as quantum cryptography [42], quantum teleportation [43] and quantum dense coding [44]. In the framework of cavity quantum electrodynamics QED, several schemes [45,46,47,48] have been proposed to generate entanglement via interaction among atoms and fields. Recently, much attention has been paid to the properties of entanglement for the models of light–matter interaction via models whose principal quantum system is composed of more than one two-level atom coupled with a single-mode field and also with each other via the dipole–dipole and Ising-like interaction [49,50], including spin–spin interaction, trapped ions [51] microcavities [52] and dipolarly coupled two molecules [53,54]. In addition, in this direction, based on the resonant two-atom JC model, an interesting application is proposed to realize a new protocol to uniquely distinguish the Bell state of two qubits [55].

However, neglecting the influence of time dependence in any quantum system cannot give a complete description of the phenomena related to that system. Then, it is more appropriate to assume the influence of time dependence when examining physical models than to consider conservative systems without time dependence [56].

In the past few years, coherent states (CSs) of harmonic oscillators and other systems have attracted widespread interest [57,58,59] and have played a vital role in various fields of quantum physics. It is known that the CSs were known as the eigenstate of the annihilation operator. Moreover, the CSs have been described as a linear combination between the Fock states [60,61]. The power-law potentials (PLPs) state depends on the eigenvalues of a Hamiltonian having a one-dimensional power-law potential [62]. More recently, the power-law potentials (PLPs) have provided many promising applications in theoretical and experimental physics, and provided the possibility to describe a large class of quantum systems [63,64]. The CSs for this kind of potentials are helpful and provide more insights in various topics [65,66,67]. Based on the above considerations, this study is devoted to explore the role of PLPs on the control of some important quantumness measures, such as von Neumann entropy, concurrence and Mandel parameter, considering the Tavis–Cummings model that describes qubits–field interaction under the effect of velocity and acceleration. The obtained results provide many different interesting phenomena that are rather significant in different tasks of quantum information and optics with optimal conditions.

The paper is organized as follows. In Section 2, we introduce the CSs of PLPs and the physical model. In Section 3, we describe the quantumness measures and explain the obtained results. A brief conclusion is given in Section 4.

## 2. Physical Model and System Dynamics

The general expression of a one-dimensional PLP is introduced as [68]

(1) $$V\left(x,k\right)=V_{o}{\left|\frac{x}{a}\right|}^{k},$$

where $V_{o}$ and *a* describe the dimensions of energy and length, respectively. *k* is a positive real number known as the power-law exponent. These PLPs can be utilized to introduce a large class of quantum systems by a proper choice of the exponent *k*.

The Hamiltonian associated to PLPs has the following expression

(2) $$\hat{H}=\frac{{\hat{p}}^{2}}{2m}+\hat{V}\left(x,k\right),$$

the corresponding eigenvalue equations are given by

(3) $$\hat{H}\left(k\right)|n\rangle =E_{n,k}|n\rangle ,\;\;\;\;\;n\geq 0.$$

The Fock states $|n\rangle$ are the eigenstates and $E_{n,k}$ are the corresponding eigenenergies.

Substituting Equation (2) into Equation (3), we obtain

(4) $${\hat{p}}^{2}|n\rangle =\left[2m(E_{n,k}-\hat{V})\right]|n\rangle ,$$

where

(5) $$p\left(x\right)=\sqrt{2m(E_{n}-V)}.$$

The eigenenergy spectrum $E_{n,k}$ can be obtained within the Wentzel–Kramers–Brillouin (WKB) approximation, such that

(6) $$\int_{-x_{o}}^{+x_{o}}p\left(x\right)dx=\left(n+\frac{g}{4}\right)\pi \hbar ,$$

where $\pm x_{o}$ are the classical turning points. Here, *g* is the Maslov index, which accounts for the boundary effects at the classical turning points, E=V(x); we then have

(7) $$\pm x_{o}=\pm a{\left(\frac{E}{V_{o}}\right)}^{\frac{1}{k}}.$$

Using Equation (1) and Equation (5), Equation (6) can be written as

(8) $$2\int_{0}^{x_{o}}\sqrt{2m\left(E_{n}-V_{o}{\left(\frac{x}{a}\right)}^{k}\right)}dx=\left(n+\frac{g}{4}\right)\pi \hbar .$$

This integral can be solved using the substitution, $u={\left(\frac{x}{a}\right)}^{k}$ with $dx=\frac{a}{k}u^{\frac{1}{k}-1}du$, and we have

(9) $$\frac{2a\sqrt{2m}}{k}\int_{0}^{\frac{E}{V_{o}}}\sqrt{\left(E_{n}-V_{o}u\right)}u^{\frac{1}{k}-1}du=\left(n+\frac{g}{4}\right)\pi \hbar ,$$

where

(10) $$\int_{0}^{\frac{E}{V_{o}}}\sqrt{\left(E_{n}-V_{o}u\right)}u^{\frac{1}{k}-1}du=\frac{1}{V_{o}^{\frac{1}{k}}}\frac{\Gamma \left(\frac{1}{k}\right)\Gamma \left(\frac{2}{3}\right)}{\Gamma (\frac{1}{k}+\frac{2}{3})}E_{n}^{\frac{1}{k}+\frac{1}{2}}.$$

Therefore, the eigenenergy spectrum is given by

(11) $$\begin{array}{ccc} E_{n,k} & = & {\left[\left(n+\frac{g}{4}\right)\pi \hbar \frac{kV_{o}^{\frac{1}{k}}}{2a\sqrt{2m}}\frac{\Gamma (\frac{1}{k}+\frac{2}{3})}{\Gamma \left(\frac{1}{k}\right)\Gamma \left(\frac{2}{3}\right)}\right]}^{\frac{2k}{k+2}},\\ & = & \omega \left(k\right){\left(n+\frac{g}{4}\right)}^{\frac{2k}{k+2}}, \end{array}$$

where $\omega \left(k\right)={\left[\frac{\pi \hbar }{2a\sqrt{2m}}V_{o}^{\frac{1}{k}}\frac{\Gamma (\frac{1}{k}+\frac{2}{3})}{\Gamma \left(\frac{1}{k}+1\right)\Gamma \left(\frac{2}{3}\right)}\right]}^{\frac{2k}{k+2}}$ is the effective frequency.

The parameter *k* determines the type of potential. To gain insight into the structure of the energy spectrum given by Equation (11), we take into account the energy difference between levels

(12) $$\begin{array}{ccc} \Delta E_{n}^{\left(k\right)} & = & E_{n}^{\left(k\right)}-E_{n-1}^{\left(k\right)}\\ & \propto & {\left(n+\frac{g}{4}\right)}^{\frac{k-2}{k+2}}. \end{array}$$

Equation (12) shows that for k=2, $\Delta E_{n}^{\left(k\right)}$ is independent on *n*, so the energy spectrum is equally spaced. For the exponent k≠2, the level spacing varies with *n*. For k>2, the energy difference increases with *n* (tightly binding potentials), whereas for k<2, the energy between adjacent levels decreases with *n* (loosely binding potentials).

The CSs associated to PLPs are defined by [65,66]

(13) $$|\xi ,k\rangle ={\left[\sum_{n=0}^{\infty }\frac{{\left|\xi \right|}^{2n}}{\Omega (n,k)}\right]}^{-\frac{1}{2}}\sum_{n=0}^{\infty }\frac{\xi^{n}}{\sqrt{\Omega (n,k)}}|n\rangle$$

where

(14) $$\Omega \left(n,k\right)=\prod_{i=1}^{n}\left[{\left(i+\frac{g}{4}\right)}^{\frac{2k}{k+2}}-{\left(\frac{g}{4}\right)}^{\frac{2k}{k+2}}\right],\;\;\;\;\Omega \left(0,k\right)=1,$$

Annihilation and creation operators, *A* and $A^{†}$, associated to PLPs act on the Fock state |n〉 as $\hat{A}|n\rangle =N_{n-1}|n-1\rangle$, ${\hat{A}}^{†}|n\rangle =N_{n}|n+1\rangle$ with $N_{n}=\sqrt{E_{n+1}-E_{0}}$.

Now, we investigate the time dependence of the two qubits that are coupled with a single-mode cavity field. Hence, the system Hamiltonian can be written as follows:

(15) $$\frac{\hat{H}}{\hbar }=\omega \;{\hat{a}}^{†}\hat{a}+\sum_{j=1}^{2}\frac{\Omega_{j}}{2}\;(|e_{j}\rangle \langle e_{j}\left|-\right|g_{j}\rangle \langle g_{j}|)+\sum_{j=1}^{2}\zeta_{j}\left(T\right)\left(\hat{a}|e_{j}\rangle \langle g_{j}|+{\hat{a}}^{†}|g_{j}\rangle \langle e_{j}|\right),$$

The operators $|e_{j}\rangle (|g_{j}\rangle )$(j=1,2) represents the excited (ground) state for the qubit. While ${\hat{a}}^{†}$ and $\hat{a}$ denote the creation and annihilation operators, ω and $\Omega_{j},j=1,2$ denote the frequencies of the cavity mode and the qubits, respectively, while $\zeta_{j}\left(T\right)$ is the time-dependent coupling between the field and the qubits. Some previous studies confirmed that the coupling parameter between the field and the qubits depends on the function of the wave number and the direction of the propagation (cosλp or sinλp), where λ denotes the wave number and *p* denotes the direction of propagator [69]. For a moving qubit, the direction of the propagation depends on the velocity and acceleration of the qubits as $p=\vartheta T^{2}+\varphi T+c,$ where ϑ,φ and *c* are the acceleration, the velocity and phase coefficients, respectively [70].

Suppose that the coupling function takes the follows form,

(16) $$\zeta_{j}\left(T\right)=\varepsilon \ell \left(T\right)\left(e^{\left(i\left(\chi \left(T\right)-\eta_{j}\left(T\right)\right)\right)}+e^{\left(-i\left(\chi \left(T\right)-\eta_{j}\left(T\right)\right)\right)}\right),\;j=1,2$$

where εℓ(T),χ(T) and $\eta_{j}\left(T\right)$ are arbitrary functions that we will define later [71]. Suppose we introduce the scaled time operators,

(17) $$\begin{array}{ccc} \hat{A} & = & \hat{a}\exp\left(i\chi (T)\right),\\ |0_{j}\rangle \langle 1_{j}| & = & |e_{j}\rangle \langle g_{j}|\exp\left(\mp i\eta_{j}\left(T\right)\right),\;j=1,2 \end{array}$$

After we apply the scaled time, the above Hamiltonian (15) becomes,

(18) $$\begin{array}{ccc} \frac{\hat{H}}{\hbar } & = & \omega \left(T\right){\hat{A}}^{†}\hat{A}+\sum_{j=1}^{2}\frac{\Omega_{j}\left(T\right)}{2}\;(|0_{j}\rangle \langle 0_{j}\left|-\right|1_{j}\rangle \langle 1_{j}|)\\ & & +\varepsilon \ell \left(T\right)\sum_{j=1}^{2}\left(e^{\left(i\left(\chi \left(T\right)-\eta_{j}\left(T\right)\right)\right)}+e^{\left(-i\left(\chi \left(T\right)-\eta_{j}\left(T\right)\right)\right)}\right)\\ & & \times \left(\hat{A}\;e^{\left(-i\left(\chi \left(T\right)-\eta_{j}\left(T\right)\right)\right)}|0_{j}\rangle \langle 1_{j}|+{\hat{A}}^{†}e^{\left(i\left(\chi \left(T\right)-\eta_{j}\left(T\right)\right)\right)}|1_{j}\rangle \langle 0_{j}|\right) \end{array}$$

where $\omega \left(T\right)=\omega +\frac{\partial \chi }{\partial T}$ and $\Omega_{j}\left(T\right)=\Omega_{j}+\frac{\partial \eta_{j}}{\partial T}.$

The interaction terms in (18) are separated into four quantities; the first two terms are $|0_{j}\rangle \langle 1_{j}|\;e^{\left(2i\left(\chi \left(T\right)-\eta_{j}\left(T\right)\right)\right)}\;$ and $|1_{j}\rangle \langle 0_{j}|\;e^{\left(-2i\left(\chi \left(T\right)-\eta_{j}\left(T\right)\right)\right)}$, while the last two terms are the fast-varying terms, so it can be neglected without loss of generality. When we take $\chi \left(T\right)=\eta_{1}\left(T\right)=\eta_{2}\left(T\right)$ the Hamiltonian (18) becomes as follows

(19) $$\frac{\hat{H}}{\hbar }=\omega \left(T\right){\hat{A}}^{†}\hat{A}+\sum_{j=1}^{2}\frac{\Omega_{j}\left(T\right){\hat{\sigma }}_{z}^{\left(j\right)}}{2}+\varepsilon \ell \left(T\right)\sum_{j=1}^{2}\left(\hat{A}\;|0_{j}\rangle \langle 1_{j}|+{\hat{A}}^{†}|1_{j}\rangle \langle 0_{j}|\right)$$

In the exact resonance case, the above Hamiltonian can be rewritten as,

(20) $${\hat{H}}_{IN}=\varepsilon \ell \left(T\right)\sum_{j=1}^{2}(\hat{A}|0_{j}\rangle \langle 1_{j}|+{\hat{A}}^{+}|1_{j}\rangle \langle 0_{j}|),$$

where $\ell \left(t\right)=\sin\left(\vartheta T^{2}+\varphi T+c\right)$ is the function that describes the time-dependent interaction.

We consider that the two qubits to be initially in the Bell state

(21) $$|\varpi_{AB}\left(0\right)\rangle =\frac{1}{\sqrt{2}}\left(|0_{1},0_{2}\rangle +|1_{1},1_{2}\rangle \right)$$

and the field takes associated with the state

(22) $$|\varpi_{F}\left(0\right)\rangle =N_{\xi ,k}\left[|\xi ,k\rangle +r|-\xi ,k\rangle \right],\;N_{\xi ,k}=\frac{1}{\sqrt{1+r^{2}+2r\langle \xi ,k|-\xi ,k\rangle }},\;\mathrm{with}\;r=0,1.$$

The value r=0 corresponds to the case of CSs for PLPs and r=1 to for even cat states. The wave function of the proposed system at T=εt>0 can be formulated as

(23) $$\begin{array}{ccc} |\varpi (T)\rangle & = & \sum_{m=0}^{\infty }\Theta_{1}\left(m,T\right)|0_{1},0_{2},m\rangle +\Theta_{4}\left(m,T\right)|1_{1},1_{2},m+2\rangle \\ & & +\left\{\Theta_{2}\left(m,T\right)|0_{1},1_{2}\rangle +\Theta_{3}\left(m,T\right)|1_{1},0_{2}\rangle \right\}|m+1\rangle , \end{array}$$

with the initial condition $|\varpi (0)\rangle =|\varpi_{F}\left(0\right)\rangle ⊗|\varpi_{AB}\left(0\right)\rangle$. The coefficients $\Theta_{j}\left(m,T\right)$ can be obtained by solving

(24) $$\begin{array}{ccc} i\frac{d\Theta (m,T)}{dT} & = & R(T)\Theta (m,T),\\ \Theta (m,T) & = & \Theta \left(m,0\right)\exp-i\int_{0}^{T}R\left(t\right)dt \end{array}$$

where

(25) $$\Theta =\left(\begin{array}{c} \Theta_{1}\\ \Theta_{2}\\ \Theta_{3}\\ \Theta_{4} \end{array}\right)\;\mathrm{and}\;R\left(t\right)=\left(\begin{array}{cccc} 0 & \varepsilon \ell \left(T\right)\sqrt{n+1} & \varepsilon \ell \left(T\right)\sqrt{n+1} & 0\\ \varepsilon \ell \left(T\right)\sqrt{n+1} & 0 & 0 & \varepsilon \ell \left(T\right)\sqrt{n+2}\\ \varepsilon \ell \left(T\right)\sqrt{n+1} & 0 & 0 & \varepsilon \ell \left(T\right)\sqrt{n+2}\\ 0 & \varepsilon \ell \left(T\right)\sqrt{n+2} & \varepsilon \ell \left(T\right)\sqrt{n+2} & 0 \end{array}\right).\;$$

The two qubits density matrix can be determined by calculating the trace over the field basis as

(26) $${\hat{\rho }}_{AB}\left(T\right)={\mathrm{Tr}}_{\mathrm{Field}}\left\{|\varpi (T)\rangle \langle \varpi (T)|\right\},$$

where the diagonal elements of the two atoms density matrix are given by

(27) $$\rho_{ll}=\sum_{m=0}^{\infty }{\left|\Theta_{l}\left(m,T\right)\right|}^{2},\;\;\;l=1,2,3,4,$$

while the off-diagonal elements satisfy $\rho_{ij}=\rho_{ji}^{*}$ and have the form

(28) $$\left(\begin{array}{c} \rho_{12}\\ \rho_{13}\\ \rho_{14}\\ \rho_{23}\\ \rho_{24}\\ \rho_{34} \end{array}\right)=\left(\begin{array}{c} \sum_{m=1}^{\infty }\Theta_{1}\left(m,T\right)\Theta_{2}^{*}\left(m-1,T\right)\\ \sum_{m=1}^{\infty }\Theta_{1}\left(m,t\right)\Theta_{3}^{*}\left(m-1,T\right)\\ \sum_{m=1}^{\infty }\Theta_{1}\left(m+1,T\right)\Theta_{4}^{*}\left(m-1,T\right)\\ \sum_{m=0}^{\infty }\Theta_{2}\left(m,t\right)\Theta_{3}^{*}\left(m,T\right)\\ \sum_{m=1}^{\infty }\Theta_{2}\left(m,t\right)\Theta_{4}^{*}\left(m-1,T\right)\\ \sum_{m=1}^{\infty }\Theta_{3}\left(m,t\right)\Theta_{4}^{*}\left(m-1,T\right) \end{array}\right).$$

According to Equation (26), we are able to study the time evolution of the qubits–field entanglement and qubit–qubit entanglement. Moreover, we use the Mandel’s parameter to discuss the quantum statistics of the field.

## 3. Quantum Quantifiers and Main Results

### 3.1. Qubits–Field Entanglement and Qubit–Qubit Entanglement

To quantify the entanglement between two qubits with the field, we use the von Neumann entropy, which is given by:

(29) $$S_{N}\left(T\right)=-\mathrm{Tr}\left[{\hat{\rho }}_{AB}\left(T\right)\ln{\hat{\rho }}_{AB}\left(T\right)\right].$$

This quantity can be evaluated through the eigenvalues of the density operator ${\hat{\rho }}_{AB}$.

To evaluate the amount of the qubit–qubit entanglement, we use the concurrence defined as

(30) $$C_{AB}=\max\left\{0,\Upsilon_{1}-\Upsilon_{2}-\Upsilon_{3}-\Upsilon_{4}\right\},$$

in which $\Upsilon_{j}$ are the eigenvalues given in decreasing order of $R=\rho_{AB}\left(\sigma_{y}⊗\sigma_{y}\right)\rho_{AB}^{*}\left(\sigma_{y}⊗\sigma_{y}\right)$, where $\rho_{AB}^{*}$ denotes the conjugate of $\rho_{AB}$ and $\sigma_{y}$ is the Pauli operator. When the two qubits are in separable state then $C_{AB}=0$. While $C_{AB}=1$ indicates that two-qubit is in a maximally entangled state.

**Figure 1:**
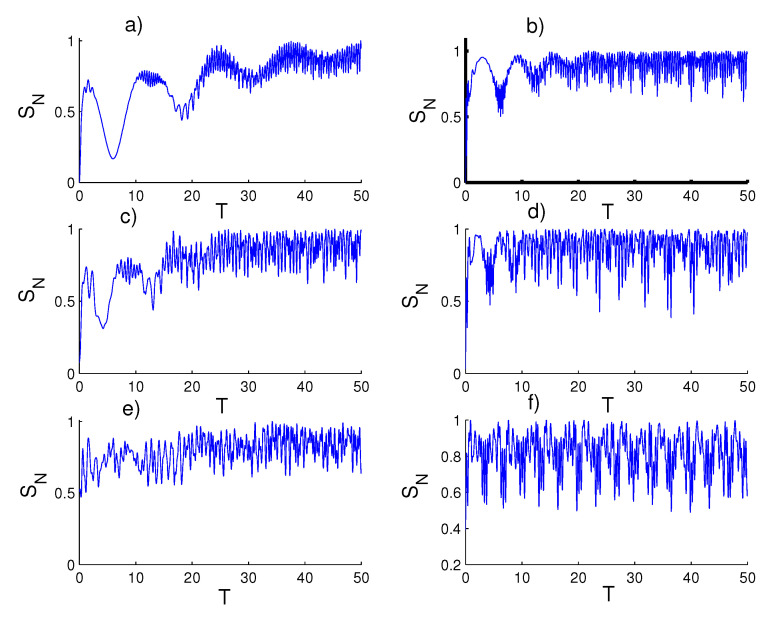
Time evolution of the von Neumann entropy $S_{N}\left(T\right)$ for $\xi =\sqrt{5}$, with the case of constant two qubits–field coupling (ϑ=φ=0 and c=π/2). Figs. (**a**,**c**,**e**) are plotted for the field initially in the coherent states (CSs) for power-law potentials (PLPs) (r=0) and Figs. (**b**,**d**,**f**) for the field initially in the SCSs for PLPs (r=1). Figs. (**a**,**b**) for harmonic well potential (k=2), Figs. (**c**,**d**) for the triangular well (k=1), and Figs. (**e**,**f**) for infinite barrier (k→∞).

In Figure 1, we display and analyze the entanglement between the two qubits and the field through the Equation (26), by setting the parameter $\xi =\sqrt{5}$ (see references [72,73]), and the other parameters (ϑ,φ,c)=(0,0,π/2). For first case, the harmonic well potential (k=2, g=2) and a coherent state (r=0), the entanglement function $S_{N}\left(T\right)$ grows gradually and reaches its peak on the revival periods, while the $S_{N}\left(T\right)$ achieves its lowest value on the collapse periods [74,75]. When setting the field in the even coherent state (r=1), the entanglement increases from the start of the interaction and the intensity of the oscillations increases. Therefore, a strong entanglement arises between the field and the two qubits, and the function does not approach the minimum value (zero) during the interaction period. For the triangular well (k=1, g=3) and a coherent state (r=0), the intensity of the oscillations decreases and the entanglement decreases slightly compared to the previous case. When the field is placed in the even coherent state (r=1), clear fluctuations are generated between the maximum and minimum values with an increase in the amplitude of the oscillations. For the infinite barrier (k→∞, g=4) and a coherent state (r=0), strong entanglement appears immediately after the beginning of the interaction with random oscillations. Moreover, the amplitude of the oscillations decreases and the $S_{N}\left(T\right)$ function does not approach minimum value (zero) during the interaction. When the even coherent state (r=1) is considered, the entanglement is weakened slightly and the $S_{N}\left(T\right)$ function regularly reaches its minimal values.

**Figure 2:**
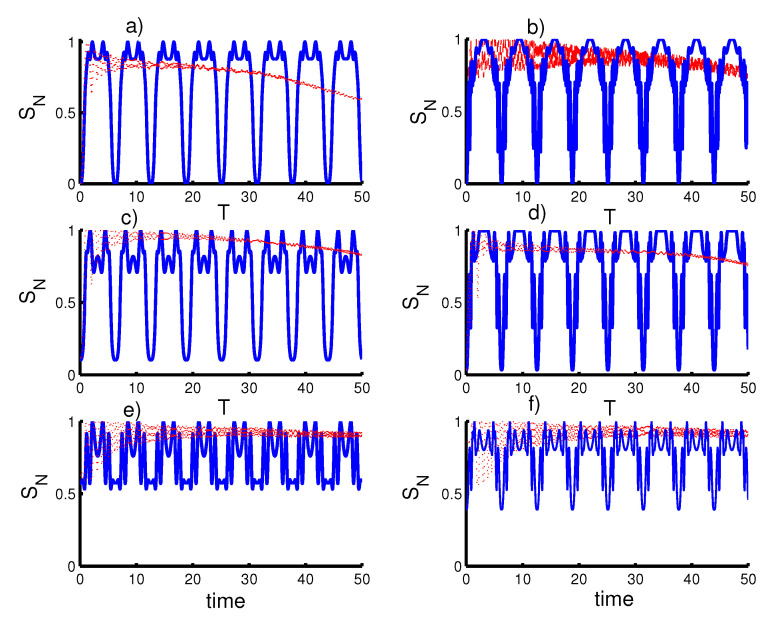
Effect of time-dependent coupling, ℓ(t), on the evolution of the atomic entropy $S_{N}\left(T\right)$ where the solid curve is for φ=1 and ϑ=c=0 (atomic speed effect) and the dotted red curve is for the acceleration effect (ϑ=φ=1 and c=0). Figs. (**a**,**c**,**e**) are plotted for the field initially in the coherent states (CSs) for power-law potentials (PLPs) (r=0) and Figs. (**b**,**d**,**f**) for the field initially in the SCSs for PLPs (r=1). Figs. (**a**,**b**) for harmonic well potential (k=2), Figs. (**c**,**d**) for the triangular well (k=1), and Figs. (**e**,**f**) for infinite barrier (k→∞).

In Figure 2, we study the effect of time dependence on the entanglement between the cavity field and the qubits with the same conditions mentioned in the first Figure. In general, when taking the velosity and the acceleration into account (ϑ=φ=1,c=0), a strong entanglement arises and the $S_{N}\left(T\right)$ function never reaches a minimum value (zero) during the interaction period. When there is only an effect of the atomic speed (ϑ=c=0,φ=1), the behavior of the $S_{N}\left(T\right)$ is completely affected. For the first case, harmonic well potential (k=2) and coherent state (r=0), strong entanglement is generated at regular intervals during the interaction time and the $S_{N}\left(T\right)$ function reaching minimum values every π. When the SCSs for PLPs (r=1) are considered, the entanglement is significantly enhanced. For the triangular well (k=1) with the field state in a coherent state (r=0), more entanglement increases periodically and the $S_{N}\left(T\right)$ function does not reach a minimum value (zero). For the field in SCSs for PLPs (r=1), $S_{N}\left(T\right)$ function tends to attain zero value. For the infinite barrier (k→∞) and the field state with r=0, the amplitude of the oscillations of $S_{N}\left(T\right)$ decreases and the entanglement between the field and the two qubits increases. In the case of the field state with r=1, the entanglement decreases slightly with increase in the amplitude of the oscillations at the smallest values of the function $S_{N}\left(T\right)$.

**Figure 3:**
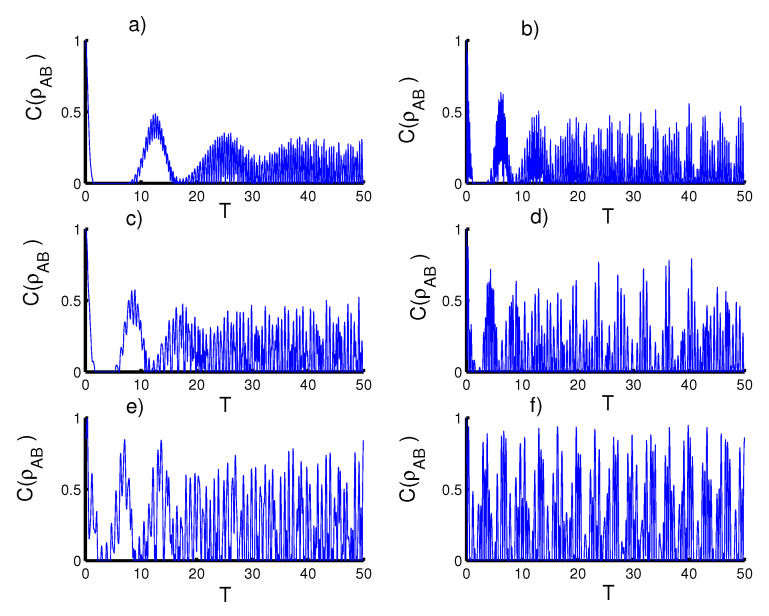
Time evolution of the the concurrence $C_{AB}\left(T\right)$ for $\xi =\sqrt{5}$, with the case of constant two qubits–field coupling (ϑ=φ=0 and c=π/2). Figs. (**a**,**c**,**e**) are plotted for the field initially in the CSs for PLPs (r=0) and Figs. (**b**,**d**,**f**) for the field initially in the SCSs for PLPs (r=1) Figs. (**a**,**b**) for harmonic well potential (k=2), Figs. (**c**,**d**) for triangular well (k=1) and Figs. (**e**,**f**) for infinite well (k→∞).

In Figure 3, we display the plots the concurrence with the same previous conditions as in Figure 1, the concurrence is considered to study the entanglement between the two qubits. For the first case, the harmonic well potential (k=2) with a coherent state (r=0), the entanglement starts from the maximaly entangled Bell state, followed by partial entanglement until the function $C_{AB}\left(T\right)$ reaches the minimum values. The phenomena of sudden death and sudden birth are achieved in multiple inverals during the interaction time. The entanglement can be enhanced after preparing the field in the even coherent state (r=1) and the periods of sudden death and sudden birth decrease. For the triangular well (k=1) with a coherent state (r=0), the intensity of the oscillations decreases and the entanglement between the qubits decreases slightly compared to the previous case. For the infinite barrier (k→∞) with a coherent state r=0, the entanglements are more powerful than the previous two cases, while the periods of sudden death and sudden birth are reduced. Moreover, the amount of entanglement between the two qubits increases and the intensity of oscillations decreases after setting the field in the even coherent state (r=1).

**Figure 4:**
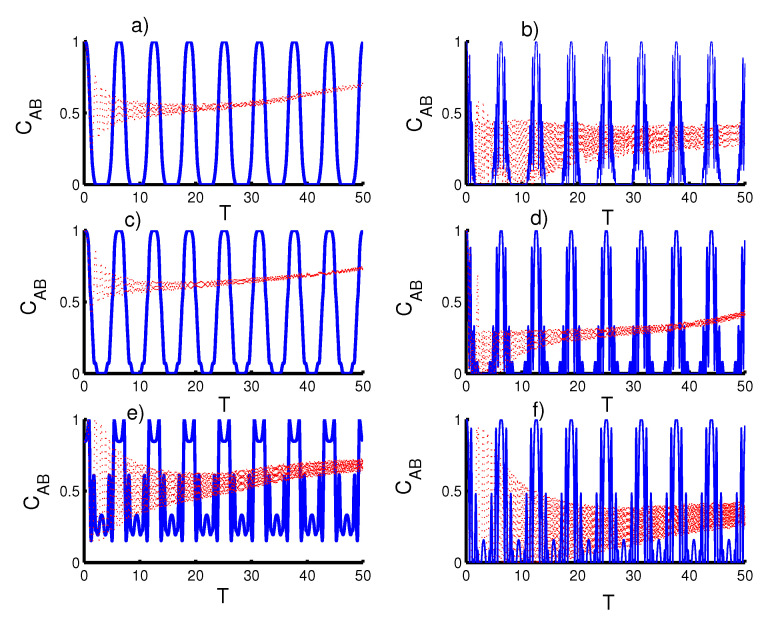
Effect of time-dependent coupling or qubit motion, ℓ(t), on the evolution of the concurrence $C_{AB}\left(T\right)$ where the solid curve is for φ=1 and ϑ=c=0 (atomic speed effect) and the dotted red curve is for the acceleration effect as (ϑ=φ=1 and c=0). Figs. (**a**,**c**,**e**) are plotted for the field initially in the coherent states (CSs) for power-law potentials (PLPs) (r=0) and Figs. (**b**,**d**,**f**) for the field initially in the SCSs for PLPs (r=1). Figs. (**a**,**b**) for harmonic well potential (k=2), Figs. (**c**,**d**) for the triangular well (k=1), and Figs. (**e**,**f**) for infinite barrier (k→∞).

In Figure 4, we study the effect of time dependence on the entanglement the qubits with the same conditions mentioned in the first figure. For the first case, k=2 with r=0 and ϑ=c=0,φ=1, the concurrence function $C_{AB}\left(T\right)$ fluctuates smoothly between the maximum and minimum values. Therefore, periods of sudden death and sudden birth are realized regularly. Note that the periods of sudden death and sudden birth increase after the inclusion of the even coherent state (r=1). In the second case (the triangular well), the entanglement function behavior the saming as the first case (the harmonic well potential), with an increase in the intensity of fluctuations. For the infinite barrier, the entanglement increases significantly to the point where the phenomenon of sudden death and sudden birth disappear. Moreover, the entanglement function reaches maximum values for every time interval 2π. The phenomena of sudden death and sudden birth returns after preparing the field in the even coherent state (r=1). For the second case of time dependence (ϑ=φ=1 and c=0), the entanglement can be enhanced for the fields with r=0, especially in the harmonic well potential case. The entanglement decreases when preparing the field in the even coherent state (r=1).

### 3.2. Photon Statistics

In order to deeper our understanding on the underlying physics of this system, we analyze the nonclassicality of the field through studying the Poissonian distribution of the photons. These properties are identified by the Mandel’s parameter [76]

(31) $$P_{M}=\frac{\langle {\hat{N}}^{2}\rangle }{\langle \hat{N}\rangle }-\langle \hat{N}\rangle -1.$$

The field photon statistics is classified according to the value of the parameter $P_{M}$ as $P_{M}>0$ and $P_{M}<0$ for the case of super-Poissonian and sub-Poissonian distribution, respectively. The Poissonian distribution is obtained in the case $P_{M}$ has zero value.

**Figure 5:**
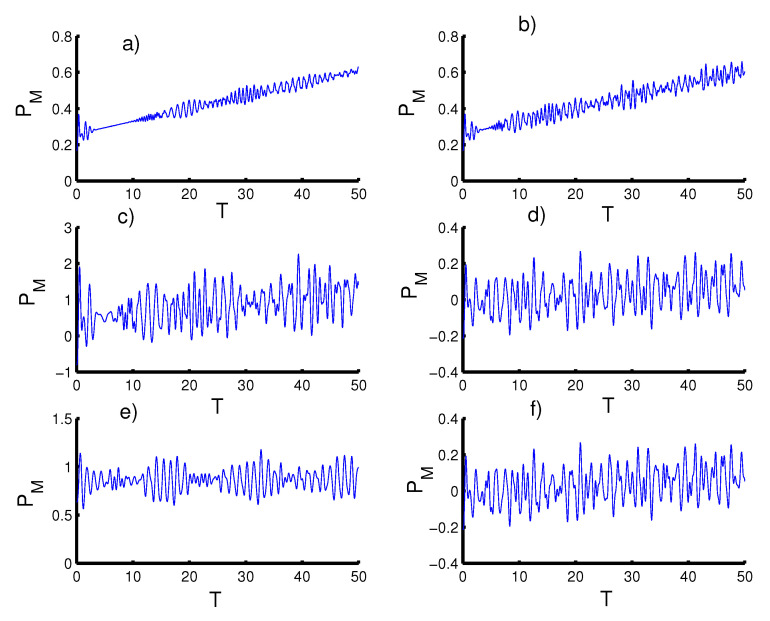
Time evolution of the Mandel parameter $P_{M}$ for $\xi =\sqrt{5}$ with the case of constant qubits–field coupling (ϑ=φ=0 and c=π/2). Figs. (**a**,**c**,**e**) are plotted for the field initially in the CSs for PLPs (r=0) and Figs. (**b**,**d**,**f**) for the field initially in the SCSs for PLPs (r=1). Figs. (**a**,**b**) for harmonic well potential (k=2), Figs. (**c**,**d**) for triangular well (k=1) and Figs. (**e**,**f**) for infinite well (k→∞).

In Figure 5, we study the Mandel parameter to define regions of the nonclassical distribution of photons. For the first case, (k=2 with r=0), the Mandel parameter $P_{M}$ indicates a super-Poissonian distribution. The Mandel parameter gradually increases with increasing time, whether the field is set in the coherent or even coherent states. The nonclassical distribution appears in the second case, k=1, with r=0, it is more pronounced when setting the field in the even coherent state (r=1). In the third case, k→∞ with r=0, the nonclassical distribution completely disappears and the super-Poisson distribution appears again.

**Figure 6:**
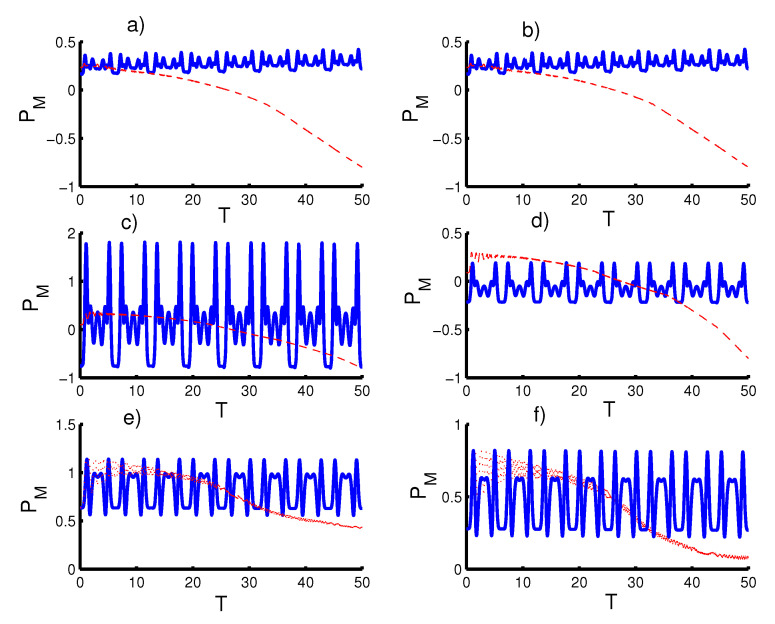
Effect of time-dependent coupling, ℓ(t) on the evolution of the Mandel parameter $P_{M}$ where the solid curve is for φ=1 and ϑ=c=0 (atomic speed effect) and the dotted red curve is for the acceleration effect as (ϑ=φ=1 and c=0). Figs. (**a**,**c**,**e**) are plotted for the field initially in the coherent states (CSs) for power-law potentials (PLPs) (r=0) and Figs. (**b**,**d**,**f**) for the field initially in the SCSs for PLPs (r=1). Figs. (**a**,**b**) for harmonic well potential (k=2), Figs. (**c**,**d**) for the triangular well (k=1), and Figs. (**e**,**f**) for infinite barrier (k→∞).

In Figure 6, we examine the influence of the time dependence on the Mandel parameter. The time dependence strongly affects the Mandel parameter. In the case of time dependence (ϑ=c=0, φ=1), we consider the first case (k=2,r=0). The Mendel parameter oscillates in the positive parts (super-Poisson distribution) and the distribution never reaches the negative regions (sub-Poisson distribution). In the second case (k=1, r=0) the nonclassical distribution (sub-Poisson distribution) appears uniformly during the interaction period. Moreover, the nonclassical distribution decreases after the inclusion of the even coherent sate of the interaction cavity. While the distribution super-Poisson appears again in the third case (k→∞, r=0), and the sub-Poisson distribution completely disappears, whether the field is set in the coherent or even coherent states. When considering the coupling dependence on time (ϑ=φ=1, c=0), we note that the nonclassical distribution appears in the first (k=2) and second (k=1) cases, while disappears in the last case (k→∞).

### 3.3. Effect of Qubit–Qubit Interaction

In this section, we examine the effect of qubit–qubit interaction parameter *D* within the interaction Hamiltonian

(32) $${\hat{H}}_{IN}=\varepsilon \ell \left(t\right)\sum_{j=1}^{2}(\hat{A}|0_{j}\rangle \langle 1_{j}|+{\hat{A}}^{+}|1_{j}\rangle \langle 0_{j}|)+D(|0_{1}1_{2}\rangle \langle 1_{1}0_{2}\left|+\right|1_{1}0_{2}\rangle \langle 0_{1}1_{2}|).$$

**Figure 7:**
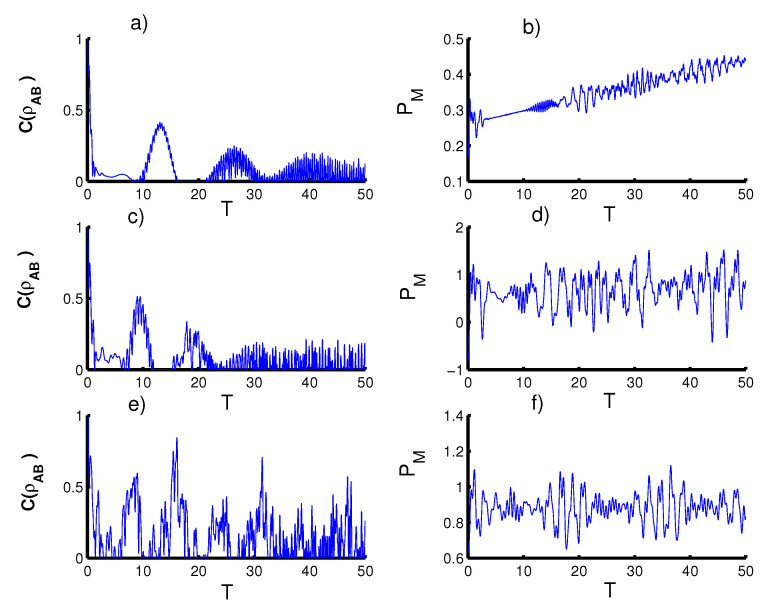
Effect of qubit–qubit interaction (D=1) in the absence of time-dependent coupling or qubit motion ($\sin(\vartheta T^{2}+\varphi T+c)$ for ϑ=φ=0 and c=π/2) for the three CSs for PLPs studied: Figs. (**a**,**b**) for harmonic well potential (k=2), Figs. (**c**,**d**) for triangular well (k=1) and Figs. (**e**,**f**) for infinite well (k→∞).

The solution of the above Hamiltonian (32) is calculated numerically. In Figure 7, we show the effect of the qubit–qubit interaction term on the entanglement and the Mandel parameter. We Note that the von Neumann entropy does not affected by adding the qubit–qubit interaction to the interaction cavity. Therefore, consider the concurrence to test the effect of the qubit–qubit interaction on the entanglement. In the first case (k=2,r=0), the amount of the entanglement between the two qubits gradually reduced with increasing time. The phenomena of sudden death and sudden birth increase. The Mandel parameter is not affected by the inclusion of the two qubits interacting together, so the Mandel parameter $P_{M}$ indicates that the super-Poisson distribution and the function $P_{M}$ does not reach the nonclassical distribution. In the second case (k=1, r=0), the entanglement $C_{AB}\left(T\right)$ decreases with increasing interaction time. Whereas in the third case (k→∞,r=0), the improvement in entanglement is more pronounced. In general, the Mandel parameter states that the distribution of photons is classical for all parameters values and the nonclassical distribution appears in short intervals for the second case (k=1, r=0).

## 4. Conclusions

In this work, we have introduced a nonlinear version of the Tavis–Cummings model for two two-level atoms interacting with a single-mode field within a cavity in the context of power-law potentials. We have considered the effect of the particle position inside the cavity field that depends on the velocity and acceleration, and the coupling parameter is supposed to be time-dependent. We have examined the effect of velocity and acceleration on the dynamical behavior of some quantumness and nonlocality measures namely; von Neumann entropy, concurrence and Mandel parameter. We have considered that the two qubits are initially prepared in the Bell states and the field from the generalized coherent states for power-law potentials state. We have examined the time evolution of entanglement of the cavity field and the two qubits through the von Neumann entropy, the entanglement between two the qubits together through the concurrence as well as the distribution of the photons through the Mandel parameter, for different potentials. We have shown in detail the dynamical behavior of the different measures for the cases of harmonic, triangular and infinite barrier potentials with respect to the main physical parameters in the absence and presence of the qubit motion. The obtained results present potential applications for quantum information and optics with optimal conditions.

## Data Availability

Not applicable.
